# Diagnostic performance of 3D automated breast ultrasound (3D-ABUS) in a clinical screening setting—a retrospective study

**DOI:** 10.1007/s00330-023-10568-5

**Published:** 2024-01-19

**Authors:** Femke Klein Wolterink, Nazimah Ab Mumin, Linda Appelman, Monique Derks-Rekers, Mechli Imhof-Tas, Susanne Lardenoije, Marloes van der Leest, Ritse M. Mann

**Affiliations:** 1 Department of Imaging, Radboud University Medical Center, Geert Grooteplein 10, P.O. Box 9101 (667), 6500 HB Nijmegen, The Netherlands; 2 Department of Radiology, Faculty of Medicine, University Teknologi MARA, Selangor, Malaysia; 3 Department of Radiology, Alexander Monro Hospital, Bilthoven, The Netherlands; 4 Department of Radiology, Netherlands Cancer Institute, Amsterdam, The Netherlands

**Keywords:** Breast cancer screening, Breast cancer, Digital breast tomosynthesis, Retrospective, Ultrasound

## Abstract

**Objectives:**

To assess the diagnostic performance of 3D automated breast ultrasound (3D-ABUS) in breast cancer screening in a clinical setting.

**Materials and methods:**

All patients who had 3D-ABUS between January 2014 and January 2022 for screening were included in this retrospective study. The images were reported by 1 of 6 breast radiologists based on the Breast Imaging Reporting and Data Systems (BI-RADS). The 3D-ABUS was reviewed together with the digital breast tomosynthesis (DBT). Recall rate, biopsy rate, positive predictive value (PPV) and cancer detection yield were calculated.

**Results:**

In total, 3616 studies were performed in 1555 women (breast density C/D 95.5% (*n* = 3455/3616), breast density A/B 4.0% (*n* = 144/3616), density unknown (0.5% (*n* = 17/3616)). A total of 259 lesions were detected on 3D-ABUS (87.6% (*n* = 227/259) masses and 12.4% (*n* = 32/259) architectural distortions). The recall rate was 5.2% (*n* = 188/3616) (CI 4.5–6.0%) with only 36.7% (*n* = 69/188) cases recalled to another date. Moreover, recall declined over time. There were 3.4% (*n* = 123/3616) biopsies performed, with 52.8% (*n* = 65/123) biopsies due to an abnormality detected in 3D-ABUS alone. Ten of 65 lesions were malignant, resulting in a positive predictive value (PPV) of 15.4% (*n* = 10/65) (CI 7.6–26.5%)). The cancer detection yield of 3D-ABUS is 2.77 per 1000 screening tests (CI 1.30–5.1).

**Conclusion:**

The cancer detection yield of 3D-ABUS in a real clinical screening setting is comparable to the results reported in previous prospective studies, with lower recall and biopsy rates. 3D-ABUS also may be an alternative for screening when mammography is not possible or declined.

**Clinical relevance statement:**

3D automated breast ultrasound screening performance in a clinical setting is comparable to previous prospective studies, with better recall and biopsy rates.

**Key Points:**

• *3D automated breast ultrasound is a reliable and reproducible tool that provides a three-dimensional representation of the breast and allows image visualisation in axial, coronal and sagittal.*

• *The diagnostic performance of 3D automated breast ultrasound in a real clinical setting is comparable to its performance in previously published prospective studies, with improved recall and biopsy rates.*

• *3D automated breast ultrasound is a useful adjunct to mammography in dense breasts and may be an alternative for screening when mammography is not possible or declined.*

**Supplementary Information:**

The online version contains supplementary material available at 10.1007/s00330-023-10568-5.

## Introduction

Breast cancer is the main cause of cancer death in the Western female population [1, 2]. Imaging plays an important role in the early detection of breast cancer, with mammography as the gold standard modality in screening [2]. Screening mammography has been shown to reduce breast cancer mortality by at least 20% [3–6].

However, mammography is less sensitive in women with dense breasts due to the overlapping fibroglandular tissue obscuring lesions. The mortality reduction due to mammography screening in women with fatty breasts is estimated to be 41%, in contrast to 13% in women with extremely dense breasts [7]. In clinical practice, supplemental ultrasound may be added for women with dense breasts to improve lesion detection.

Supplemental ultrasound detects significantly more invasive breast cancers at an early stage in women with dense breasts [8–10]. Targeted 2D hand-held ultrasound (HHUS) is implemented as an additional study within some screening programnmes [10, 11]. The limitations of HHUS include operator dependency and variability in acquisition, which reduces reproducibility [10–12].

3D automated breast ultrasound (3D-ABUS) has been implemented in clinical practice for several years. In 3D-ABUS, three overlapping images are taken per breast and can be evaluated in a multiplanar format [11, 13]. Hence, the entire breast between the midsternal and midaxillary lines is acquired and recorded. With 3D-ABUS, the standardised scan can be performed by medical personnel other than a radiologist. Although there is increased reading time, the radiologists’ workload is ultimately reduced, allowing work efficiency in image interpretation and diagnosis [11, 13, 14]. Further benefits of 3D-ABUS include the ability to evaluate the whole breast with increased reproducibility, allowing a more accurate temporal image comparison [11, 15]. Additional benefits of 3D-ABUS include the availability of coronal plane, as lesions may be identified in one of the 3 planes [16]. 3D-ABUS is also reported to outperform HHUS in detection of architectural distortion from the coronal plane [17, 18].

In our department, screening digital breast tomosynthesis (DBT) is offered to women at intermediate risk yearly from age 40, and biannually for women aged 50–75 at average risk of breast cancer. In women with dense breast tissue, 3D-ABUS is added as a supplementary examination. After review of the DBT and 3D-ABUS, if necessary, a targeted HHUS is offered. Magnetic resonance imaging (MRI) and mammography as screening is offered for high-risk patients (lifetime risk > 20% (Tyrer-Cuzick)), and these women are excluded from 3D-ABUS screening [19].

Several prospective studies have reported an additional cancer detection between 1.9 and 7.7 per 1000 women screened with 3D-ABUS [11, 13, 14, 20]. Aside from an increase in sensitivity, a decrease in specificity with an increase in the recall rate and biopsy rate has been reported, with the published recall rate ranging from 5.4 to 28.5% [13–15, 20]. The biopsy rate was also reported to increase by 3.6 and 7.2% [13, 15].

Although an increase in cancer detection yield in women with dense breasts with the additional use of 3D-ABUS in screening has been reported in previous prospective trials [13–15, 20], it is not yet known how the results translate into real clinical practice, where reported recall rates are higher than what is generally accepted in European practice. The research questions posed in the previous studies were to answer whether 3D-ABUS could improve breast cancer detection and may therefore have shifted the balance between sensitivity and specificity. The added value of supplemental 3D-ABUS may differ in real clinical practice. A retrospective study over a longer period of time may address this question. Henceforth, in this study, we aimed to assess the breast cancer detection yield, predominantly in screening of women with dense breasts, with a minority of cases in the non-dense women, in our clinical routine.

## Methods

### Study design

This retrospective study was approved by the institutional review board and the need for informed consent from the patients was waived.

This study included all patients who underwent 3D-ABUS imaging of the breasts for screening between January 2014 and January 2022. In essence, 3D ABUS was offered to all women who had a clinical screening indication and dense breasts on mammography or DBT (category C and D) or by request of the patient (including follow up of histopathology-proven benign lesions)). The breast density was assessed from the digital mammogram/DBT using Volpara software. Women with an indication for supplemental breast MRI were excluded. At the discretion of the attending radiologist, some women with Volpara breast density A and B (non-dense) were also offered 3D-ABUS following visual BI-RADS density assessment. In addition, 3D ABUS was offered for screening in women who refused DBT or were younger than the mammography-recommended age group. Diagnostic cases were excluded. Patient’s age, imaging indication, Breast Imaging and Data Reporting System (BI-RADS) score, breast density category, patient’s outcome, investigations and their results were assessed from the electronic medical record (EMR) and were recorded in an Excel worksheet. The follow-up studies included targeted HHUS, magnetic resonance imaging (MRI) and core needle or vacuum-assisted biopsies. The histopathology results were collected from the EMR.

### Patient population

**Fig. 1:**
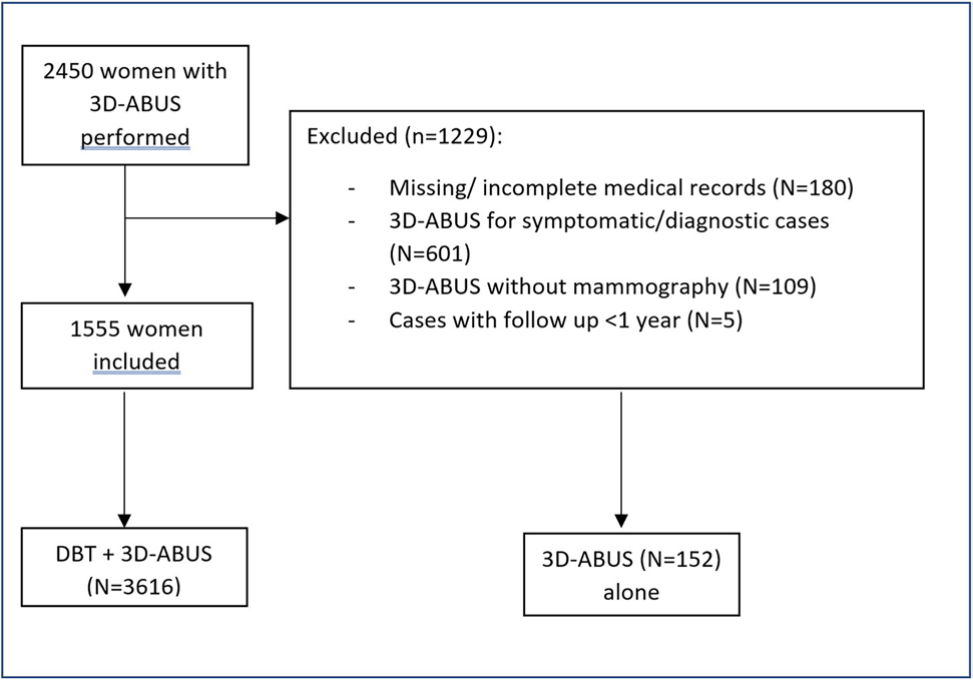
Flow chart diagram of the study population

**Fig. 2:**
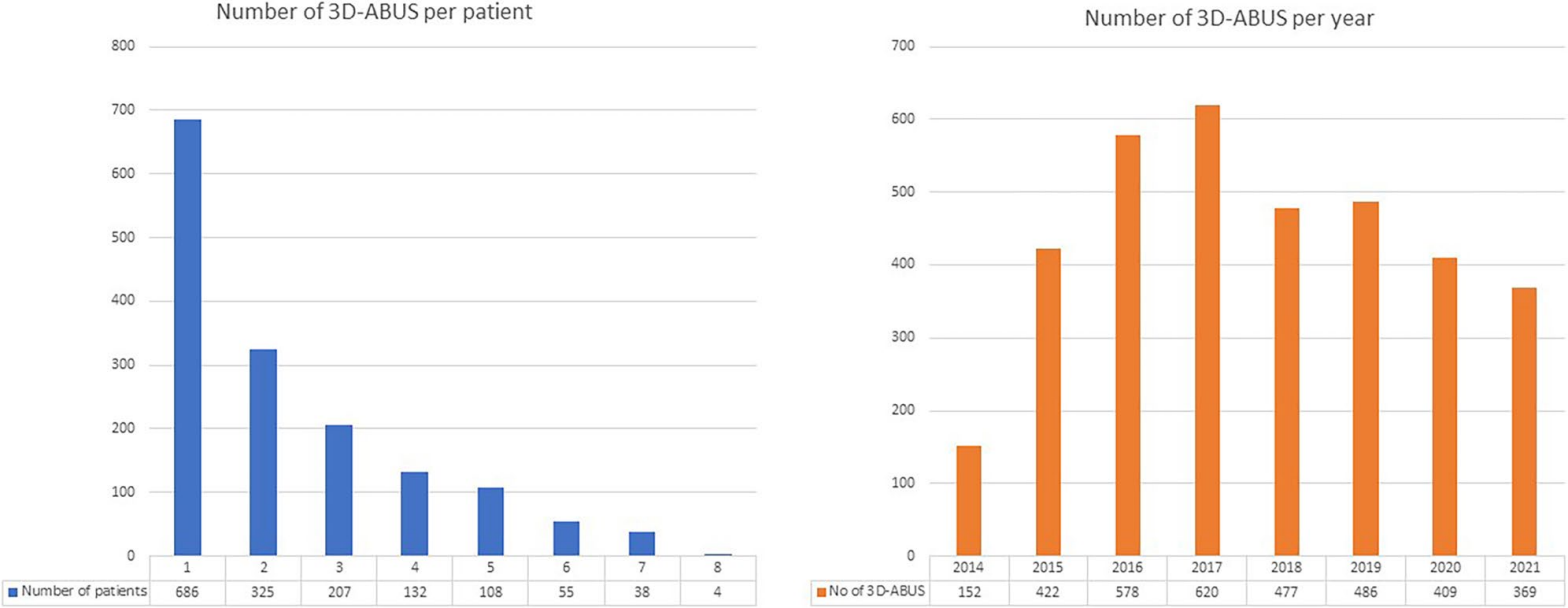
Number of 3D-ABUS per patient in the study population and number of 3D-ABUS examinations throughout years from 2014 to 2021

During the study period, 3D-ABUS was performed on 2450 women. Of these, 1555 women aged between 30 and 87 (mean 55.3) underwent the examination primarily for screening and were included in this study. Figure 1 shows a flow chart diagram of the study population with the exclusion criteria. Patients younger than 40, who were included, had a family history of breast cancer and/or atypia (surgically excised) detected in a previous biopsy. There was a total of 3616 3D-ABUS examinations performed during the study period, which amounted to an average of 2.35 examinations per patient (range 1 to 8). Figure 2 is showing the number of patients within each screening round. All these studies were DBT with 3D-ABUS.

However, there were 152 examinations in 104 women with only 3D-ABUS performed without mammography (age range of 18–86, mean 49.6). This group of patients was analysed separately.

Table 1 shows the demographic characteristics of the study population.

**Table 1:** Screening indication and breast density of the study population

Indication for screening at the initial presentation	% (*N*)
Family history of breast cancer	37.3% (580)
Personal history of breast cancer	35.6% (553)
Gene mutation	2.7% (42) 59.5% (25) BRCA-1/2, 11.9% (5) NF1, 23.8% (10) CHEK2, 4.8% (2) CDH1*
Follow-up for benign findings^†^	12.6% (196)
Previous history of chest-wall radiation therapy	1.1% (17)*
Age group outside of the population screening	5.5% (85)
Others	5.3% (82)
Total	100% (1555)

^*^In the Netherlands, these women are offered MRI screening until the age of 60. Thereafter, they revert to annual mammographic screening, in our centre supplemented with 3D ABUS when the breasts are still dense

^†^Benign findings from previous screening; for example, cysts and fibroadenomas

^$^The indication for 3D-ABUS was made by the radiologists and therefore sometimes overruled the automated density assessment. There were 4.0% (*n* = 144/3616) in this case

### Acquisition technique

The 3D-ABUS was performed by a trained radiographer using Siemens Acuson s2000 Automated Breast Volume Scanner Ultrasound System. This system creates 3D volumetric images using a wide linear array transducer (14L5 transducer). The whole breast was imaged in 3 to 5 acquisitions (anterior, lateral, medial, inferior and superior) depending on the breast’s size. If abnormalities detected on the 3D-ABUS warranted further investigation, a targeted HHUS was performed by a radiologist using the Siemens Acuson s2000 system with the 18 MHz 18L6 linear array transducer.

Corresponding DBT exams in standard views (craniocaudal (CC) and mediolateral oblique (MLO)) were acquired by trained radiographers using a Mammomat Revelation or Mammomat Inspiration mammography machine (Siemens).

### Image review

All studies were reviewed by one of six breast radiologists (6 to 30 years of experience in breast imaging) in almost equal distribution according to the Breast Imaging Reporting and Data System (BI-RADS) [21]. All the radiologists underwent a 2-day training in application and reading of 3D-ABUS prior to commencement of the machine in the department, which was held in year 2014 (the first year of 3D-ABUS operation). The DBT and 3D-ABUS were evaluated in the same setting, with the DBT reviewed initially, followed by the 3D-ABUS. If the patient had a previous 3D-ABUS study performed, comparison with this study was performed. When logistically possible images were reviewed whilst the patient was still present in the hospital, giving the possibility to immediately perform additional investigations if required, all images were read on the day of acquisition. When image interpretation gave rise for further evaluation in women who had already left the hospital, they were recalled for further assessment on another date. All patients with lesions scored as BI-RADS 3, 4 or 5 had subsequent HHUS. Patients with a BI-RADS score of 4 or 5 underwent a biopsy for tissue diagnosis. Patients with a BI-RADS score of 3 either underwent a biopsy or were followed up in 3–6 months. Stable lesions for 2 years were considered benign findings. Cases with follow-up less than 1 year or missing follow-up were excluded (*n* = 5).

Abnormalities detected in DBT were classified based on the ACR-BIRADS categories: ‘mass’, ‘calcifications’, ‘asymmetry’ or ‘architectural distortion’ [21]. Abnormalities detected on 3D-ABUS were divided into ‘mass’ or ‘architecture distortion’. The histopathology results were categorised as ‘malignant’ or ‘benign’.

### Statistical analysis

All statistical analyses were performed using statistical software (IBM SPSS version 25). Descriptive statistics were used. The recall rate was defined as the percentage of examinations where the patient had to return for further investigation. The biopsy rate was defined as the percentage of biopsies performed and the positive predictive value (PPV) was defined as the percentage of a malignant result per biopsies performed. The cancer detection rate was defined as the number of studies that led to the detection of breast cancer per 1000 screening studies. 95% confidence intervals (CI) of the results were calculated. Logistic regression was performed to assess the pattern of recall rate, biopsy rate and malignancy rate, with *p* value < 0.05 regarded as statistically significant.

## Results

### Abnormal findings on 3D-ABUS

In total, 3616 studies were performed in 1555 women (mean age 55.3, range 30–87) within the regular screening programme. In 259 studies, there were 87.6% (*n* = 227/259) masses and 12.4% (*n* = 32/259) architectural distortions detected. In 25.8% (*n* = 67/259) of cases, the abnormalities were seen in both the DBT and the 3D-ABUS.

### Recall rate

A total of 8.2% (*n* = 296/3616) targeted HHUS and 0.2% (*n* = 6/3616) MRIs were performed following mammography and 3D-ABUS. For abnormalities detected on 3D-ABUS and DBT, there were 1.3% (*n* = 46/3616) targeted HHUS performed. An additional 5.1% (*n* = 184/3616) targeted HHUS were performed for abnormalities seen on the 3D-ABUS alone. There was only one case recalled for an MRI instead of an HHUS. Three women were recalled for targeted HHUS but were lost to follow-up (0.1% (*n* = 3/3616)). The overview of recalls by modality is provided in the Supplementary material. There were 5.8% (*n* = 209/3616) exams rated as BI-RADS 3. Of these, 29.2% (*n* = 61/209) underwent HHUS.

**Fig. 3:**
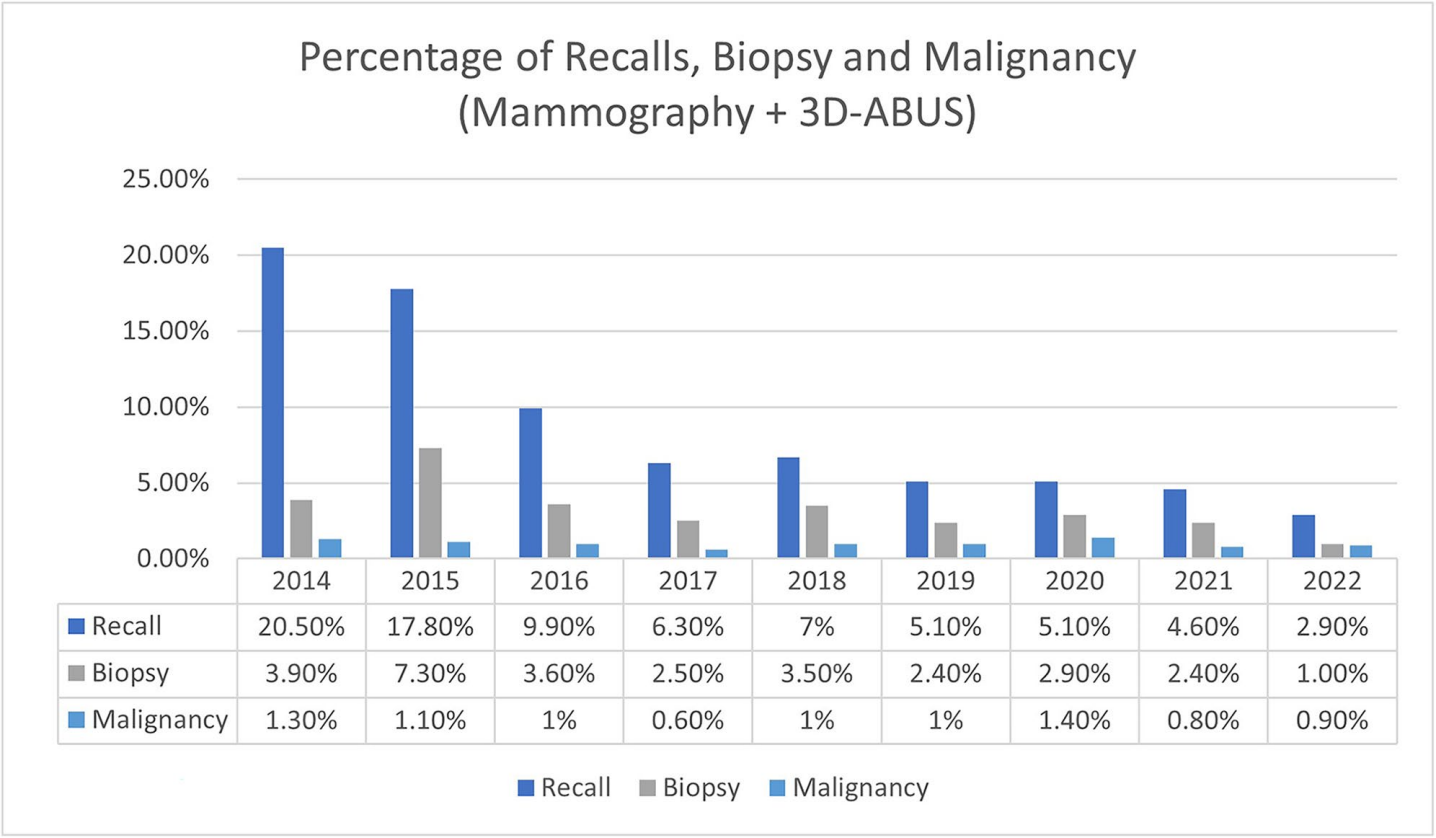
Percentage of recalls, biopsies, and malignant cases for DBT and 3D-ABUS group throughout the study from 2014 to 2022

**Fig. 4:**
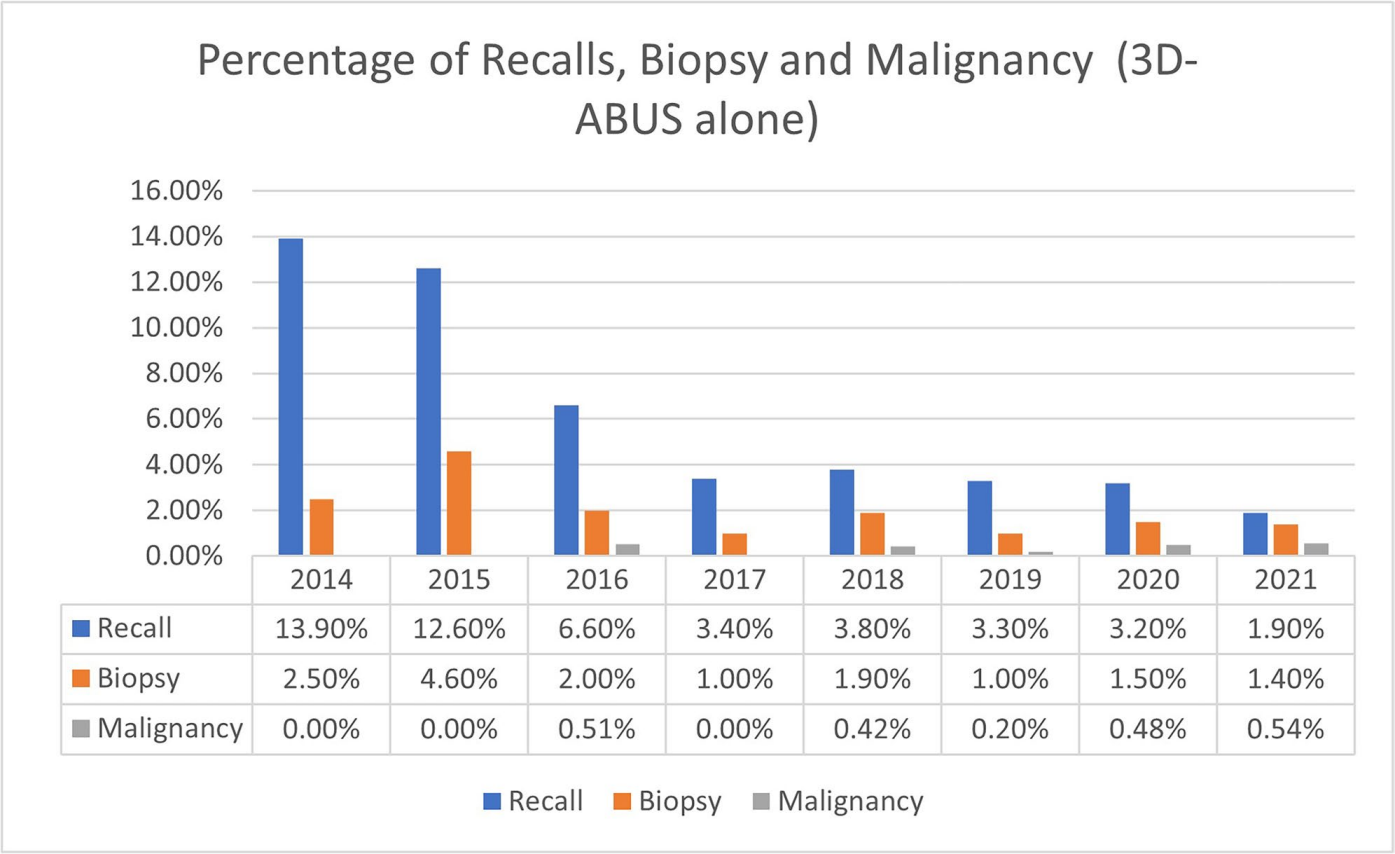
Percentage of recalls, biopsies, and malignant cases for 3D-ABUS alone throughout the study from 2014 to 2021

Over the 8 years, there was a steady decline in the recall rate for 3D-ABUS findings in both DBT + 3D-ABUS and 3D-ABUS alone (Figs. 3 and 4). Logistic regression was performed to assess the recall rate pattern throughout the years, and we noted that there was a significant decreasing pattern of 2.0% every year for DBT + 3D-ABUS (*p* = 0.001) and 1.7% every year for 3D-ABUS alone (*p* = 0.003). In the first year (2014), 23.6% (*n* = 31/151) of cases were recalled in comparison to 4.6% (*n* = 17/369) in 2021 and 3.8% (*n* = 3/103) in year 2022.

### Biopsies and histopathology results

In total, 123 biopsies were performed in 3616 studies. 76.4% (*n* = 94/123) were ultrasound-guided core needle biopsy and 23.6% (*n* = 29/123) were vacuum-assisted tomosynthesis-guided biopsy. There were 30.1% (*n* = 37/123) malignant and 69.9% (*n* = 86/123) benign cases. The percentage of biopsied cases over the years in the DBT + 3D-ABUS and 3D-ABUS alone cohorts is shown in Figs. 3 and 4. Logistic regression analysis noted a significant reduction of biopsy rate of 0.5% yearly for DBT + 3D-ABUS (*p* = 0.025). No significant reduction of biopsy rate for 3D-ABUS alone was observed (*p* = 0.095). The malignancy detection rate in both cohorts were constant throughout the years at 0.6–1.4% (*n* = 20/30) and 0.0–0.5% (*n* = 10/65), respectively (*p* = 0.525 and *p* = 0.067).

In 52.8% (*n* = 65/123) cases, the biopsy was performed due to an abnormality detected in 3D-ABUS alone, without any abnormality detected on the DBT. This results in a biopsy rate of 1.8% (CI 1.4–2.3%). In this group, there were 84.6% (*n* = 55/65) benign and 15.4% (*n* = 10/65) malignant lesions. The supplemental cancer detection rate of 3D-ABUS is therefore 2.77 per 1000 screening tests (CI 1.30–5.1). The positive predictive value (PPV) is 15.4% (*n* = 10/65) (CI 7.6–26.5%). Table 2 shows an overview of the biopsies performed.

**Table 2:** Biopsied cases and the histopathology results (*n* = 123), *N* (%)

	Ultrasound-guided biopsy		Tomosynthesis guided biopsy		
	Malignant	Benign	Malignant	Benign	Total
3D-ABUS Alone	10 (8.1%)	55 (44.7%)	0 (0%)	0 (0%)	65 (52.8%)
3D-ABUS and DBT	19 (15.4%)	10 (8.1%)	1 (0.8%)	0 (0%)	30 (24.4%)
DBT Alone	0 (0%)	0 (0%)	7 (5.7%)	21 (17.1%)	28 (22.8%)
Total	29 (23.6%)	65 (52.8%)	8 (6.5%)	21 (17.1%)	123

### Malignant tumours

**Fig. 5:**
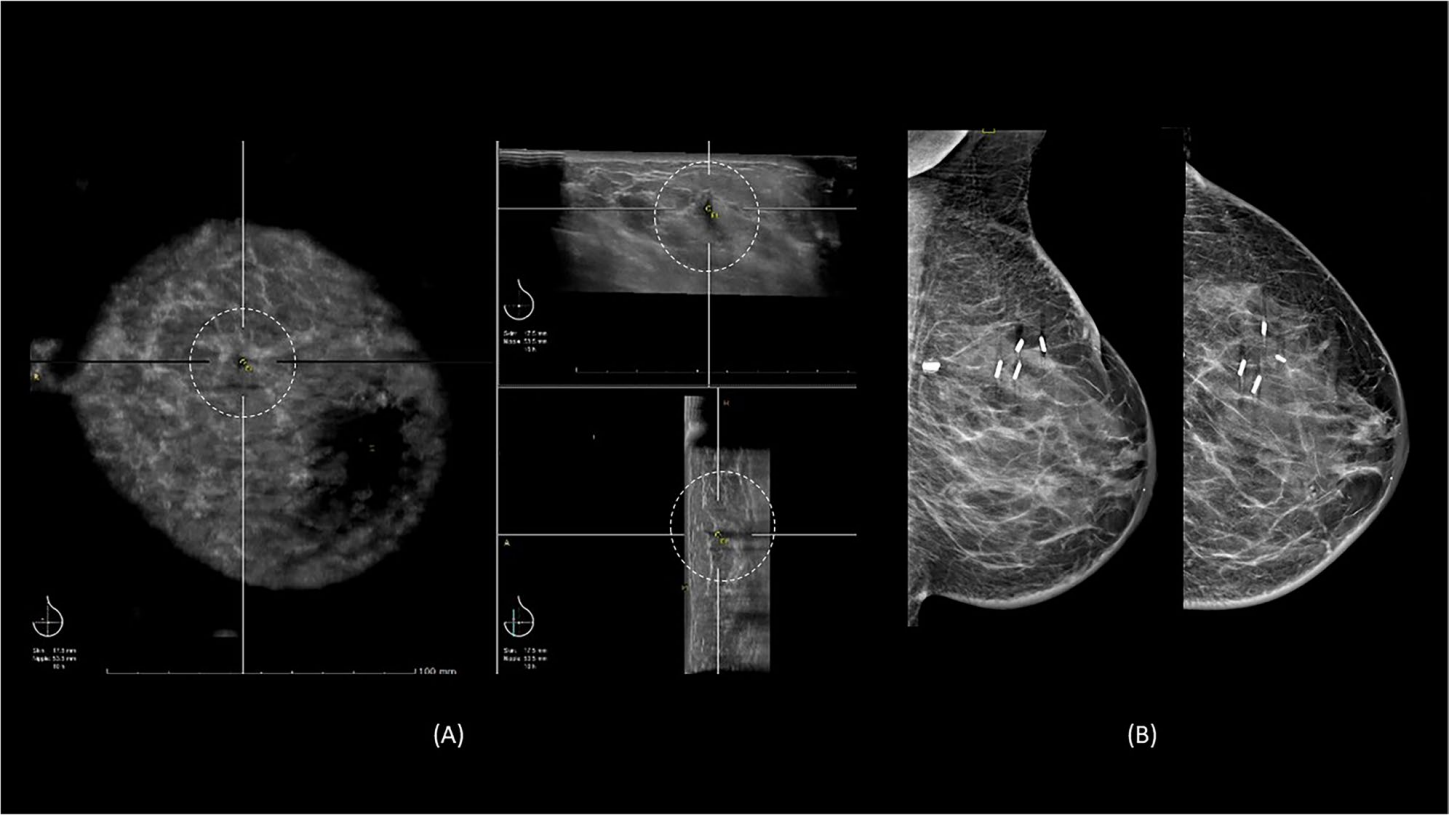
A 60-year-old woman with a history of breast cancer in the left breast. **A** There is an 8-mm lesion with architectural distortion in the left breast upper inner quadrant on 3D-ABUS (dashed circle). **B** Mammography of the left breast noted BI-RADS density C with surgical clips from previous surgery and no suspicious lesion was detected. Ultrasound-guided biopsy of the lesion confirmed invasive carcinoma NST, oestrogen receptor positive (50% positive), progesterone receptor, and HER-2 negative

**Fig. 6:**
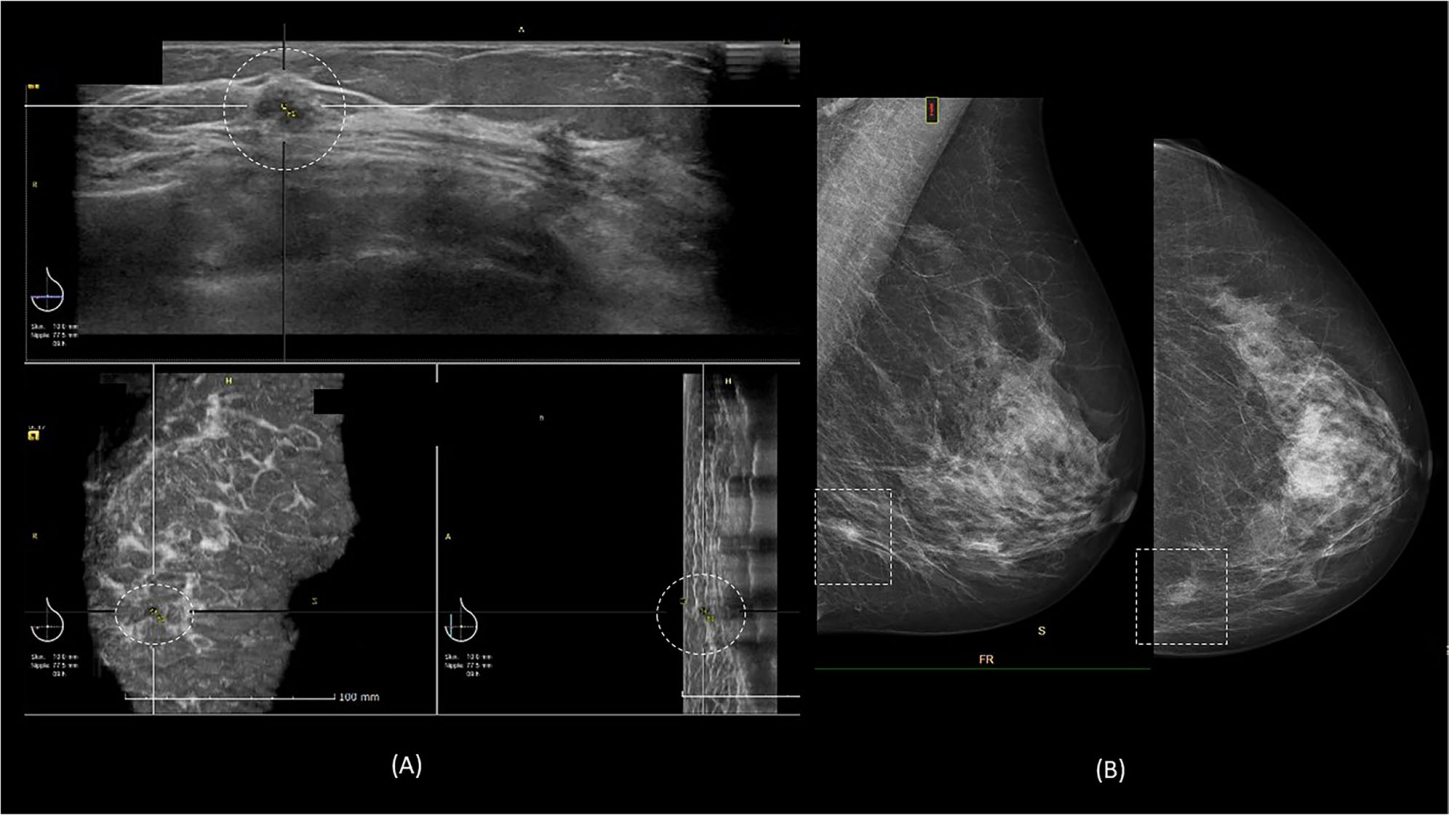
A 70-year-old woman with a family history of breast cancer. **A** 3D-ABUS noted an ill-defined and lobulated hypoechoic lesion (dashed circle) measuring 10 mm at the left mid-inner region. **B** The left mammography noted BI-RADS density C with an irregular and ill-defined equal density mass at the left lower inner quadrant (dashed box). The lesion was biopsied under ultrasound guidance, with histopathology results of invasive papillary carcinoma, oestrogen-receptor positive, progesterone receptor, and HER-2 negative

Malignancy was detected in 2.4% (*n* = 37/1555) of patients in the study population with an age range between 43 and 78 years (mean 63.1, SD 10.2). There were 27% (*n* = 10/37) discovered on the 3D-ABUS alone and 54% (*n* = 20/37) using the 3D-ABUS in combination with DBT. Figure 5 is a case example of a malignant lesion that was detected by 3D-ABUS alone, and Fig. 6 is a case example of a malignant lesion detected in DBT and 3D-ABUS. The remaining 19% (*n* = 7/37) of cases were only visible on the DBT as suspicious calcifications, with 6 cases of histopathology-proven ductal carcinoma in situ (DCIS) and one case of IDC. Table 3 provides an overview of the malignant tumour cases detected in 3D-ABUS alone, 3D-ABUS and DBT and DBT alone.

**Table 3:** Characteristics of malignant tumours detected specific to modalities

Characteristics	ABUS alone *N* (%) *n* = 10	ABUS and DBT *N* (%) *n* = 20	DBT alone, *N* (%) *n* = 7
Screening indication			
Family history of breast cancer	2 (20%)	7 (35%)	4 (57%)
Personal history of breast cancer	3 (30%)	6 (30%)	2 (29%)
Follow-up for benign findings	3 (30%)	6 (30%)	1 (14%)
Age group outside of the population mammography screening	1 (10%)	0 (0%)	0 (0%)
Others (hormone replacement therapy)	1 (10%)	1 (5%)	0 (0%)
Breast density			
A	0 (0%)	1 (5%)	0 (0%)
B	0 (0%)	2 (10%)	1 (14%)
C	7 (70%)	12 (60%)	3 (43%)
D	3 (30%)	5 (25%)	3 (43%)
Breast cancer type			
Invasive carcinoma NST	7 (70%)	14 (70%)	1 (14%)
Ductal carcinoma in situ (DCIS)	1 (10%)	1 (5%)	6 (86%)
Invasive lobular carcinoma	1 (10%)	4 (20%)	0 (0%)
Apocrine carcinoma	1 (10%)	0 (0%)	0 (0%)
Papillary carcinoma	0 (0%)	1 (5%)	0 (0%)
Bloom Richardson grading			
1	5 (50%)	7 (35%)	0 (0%)
2	2 (20%)	6 (30%)	1 (14%)
3	1 (10%)	4 (20%)	0 (0%)
Unknown	1 (10%)	2 (10%)	0 (0%)
DCIS grade			
2	0 (0%)	0 (0%)	3 (43%)
3	1 (10%)	1 (10%)	3 (43%)
Oestrogen receptor positive	8 (80%)	14 (70%)	1 (14%)
Progesterone receptor positive	5 (50%)	12 (60%)	0 (0%)
HER-2 positive	0 (0%)	2 (10%)	0 (0%)
Axillary lymph node positive	1 (10%)	5 (25%)	0 (0%)

The mean tumour size of cancers detected by 3D-ABUS alone was 11.8 mm (SD 5.6, range 4.8–23.0) with median 12.0 mm (IQR 8.0 mm) compared to DBT alone which was 30.9 mm (SD 2.4, range 0.9–8.1) with median 30.0 mm (IQR 22.0 mm). Mean tumour size of cancers detected in 3D-ABUS and DBT was 19.7 mm (range 8.0–37.0) with median 18.0 mm (IQR 15.8).

### 3D-ABUS in patients without mammography

Within the screening population, some patients underwent only 3D-ABUS without DBT. Reasons include the inability to perform mammography, a patient’s age younger than 30, or the patient’s refusal of DBT. There were 152 3D-ABUS studies performed alone on 104 patients (mean age 49.6, SD 16.4, range 18–86), with an average number of studies of 1.49 per patient (range 1 to 6). In this group, a total of 11 targeted HHUS were carried out for an abnormality detected on 3D-ABUS. The recall rate in this group of patients was 7.2% (*n* = 11/152) (CI 3.7–12.6%).

Four biopsies were performed due to an abnormality detected on 3D-ABUS. Two of these were benign and two were malignant (both invasive carcinoma NST). The biopsy ratio was 2.6% (*n* = 4/152) (CI 0.7–6.6%). The PPV was 50.0% (*n* = 2/4) (CI 6.7–93.2%).

The additional cancer detection rate for this group is 13.2 per 1000 screening studies (CI 1.6–46.7). Table 4 lists the recalls, biopsies, PPV and additional cancer detection rate in the screening population that has undergone both DBT and 3D-ABUS, as well as the screening population with 3D-ABUS alone.

**Table 4:** Recalls, biopsies, PPV and additional cancer detection in the screening population with both supplemental 3D-ABUS and in women screened with 3D-ABUS alone

	3D-ABUS and DBT (*N* = 3616)		3D-ABUS Alone (*N* = 152)	
	Number	Percentage	Number	Percentage
Recall	188	5.2% (CI: 4.49–5.97)	11	7.2% (CI: 3.67–12.58%)
Biopsy	65	1.8% (CI: 1.39–2.29)	4	2.6% (CI: 0.72–6.60%)
Positive predictive value	10	15.4% (CI: 7.63–26.48)	2	50.0% (CI: 6.7–93.24%)
Additional cancers detected	10	2.77 per 1000 examinations (CI: 1.30–5.1)	2	13.2 per 1000 examinations (CI: 1.6–46.7)

## Discussion

In this study, cancer detection with 3D-ABUS as a supplemental study to DBT was investigated in a real clinical setting. We found that the supplemental cancer detection rate is 2.77 per 1000 cases. The recall rate is 5.2%, the biopsy rate is 1.8% and PPV is 15.4%.

The rate of breast cancer detection in our study is in line with previous prospective studies that reported supplemental cancer detection of between 1.9 and 7.7 per 1000 screened women [13–15, 20]. The recall rate in our study is, however, lower compared to the previously reported prospective studies. Previous studies have reported an increased recall rate of between 5.4% and 13.4%, whilst our study noted a supplemental recall rate of 5.2% [13–15]. We also noted a steady decline of the recall rates through the years, whereas cancer detection rates remained stable. Furthermore, more than half of the cases recalled for additional examination were performed immediately following the 3D-ABUS, and thus did not require the patient to return for an additional investigation. Previous studies also reported an increase in biopsy rate in the range of 3.6 and 7.2% [13, 15]. We found that our biopsy rate was lower, at 1.8%.

A possible explanation is that previous studies were conducted within one or fewer screening rounds. The lack of comparison images at the beginning of the 3D-ABUS application may cause unnecessary recalls for subtle findings [11]. The high recall rate during the beginning of the 3D-ABUS implementation is likely also due to the learning curve for radiographers and radiologists in the execution and assessment of the study [14]. In contrast to the previous publications, in our study, the 3D-ABUS was evaluated over a period of approximately 8 years. With more screening rounds, the experience of radiographers and radiologists grew, and previous images of the same patient are available for comparison. Henceforth, the number of recalls declined over time.

In our study, the cancer detection rate is 13.2 per 1000 exams in patients who underwent 3D-ABUS alone for screening. Although the application of 3D-ABUS is proposed as an adjunct to mammography/DBT, in some cases, when patients refuse or are unable to undergo mammography, 3D-ABUS may thus be used as an alternative screening method, in specific cases. Although in this group the cancer detection rate is high, the results are less reliable due to the small sample size and warrant further investigation. However, the recall rate (7.2%) in this group is almost similar to the cohort of patients screened with both DBT and 3D-ABUS.

The majority of cancers that were detected by 3D-ABUS alone were 70% IDC (*n* = 7), whereas cancers that were detected by DBT alone were predominantly DCIS (86% (*n* = 6)). This indicates that 3D-ABUS yielded more biologically significant cancers, despite missing DCIS. The cancer size detected was smaller in 3D-ABUS alone cohort than in the DBT alone. However, the lesions detected in DBT were mostly calcifications. Henceforth, the application of 3D-ABUS is recommended in combination with DBT, in order not to miss calcified lesions.

A strong point in this study is that all patients who had a 3D-ABUS in the screening cohort were included. As a result, the largest possible research population within this institution was included, contributing to the reliability of the research. The result from our study complements the existing body of knowledge on breast cancer detection by 3D-ABUS, by showing the longitudinal performance in a clinical setting.

We acknowledge several limitations in our study. Firstly, our study analysed the practice in a single institution. As a result, the results may not be fully reflective of other clinical settings and hospitals. The same applies to the ultrasound system utilised, whereby we have only tested one system. Hence, the results from our study may differ when another 3D-ABUS system is used. Further studies using other/multiple 3D-ABUS systems in a multicenter setting may extend the assessment of the 3D-ABUS application in clinical practice. Due to the retrospective nature of the study, there remains potential patient selection bias as we cannot exclude that in some women the DBT was evaluated before the ABUS was performed and subsequently ABUS was foregone in favour of direct targeted handheld ultrasound. This implies that our study mainly reports the added value of ABUS in women with—at first glance—negative mammography evaluations. We also did not have data on interval cancer.

In conclusion, the breast cancer detection rate of 3D-ABUS in women in a real clinical setting is similar to the results reported in previous prospective studies. In addition, the recall and biopsy rates are more favourable than previously reported. Although the combination of mammography + 3D-ABUS is recommended, however, in women unable to undergo, or refusing mammography, 3D-ABUS may provide a possible solution for screening.

### Supplementary Information

Below is the link to the electronic supplementary material.

Supplementary file1 (PDF 140 KB)

## Data Availability

Data generated or analysed during the study are available from the corresponding author by request.

## Abbreviations

3D-ABUS: 3D automated breast ultrasound
BI-RADS: Breast Imaging and Data Reporting System
CC: Craniocaudal
CI: Confidence interval
DBT: Digital breast tomosynthesis
DCIS: Ductal carcinoma in situ
HHUS: Hand-held ultrasound
MLO: Mediolateral oblique
NST: No special type
PPV: Positive predictive value

## Declarations

### Guarantor

The scientific guarantor of this publication is Ritse Mann.

### Statistics and biometry

No complex statistical methods were necessary for this paper.

### Study subjects or cohorts overlap

No study subjects overlap.

### Methodology

• retrospective

• observational

• performed at one institution
