# Supplementary material for: Diagnostic performance of 3D automated breast ultrasound (3D-ABUS) in a clinical screening setting—a retrospective study
Source: Eur Radiol. 2024 Jan 19;34(8):5451–60. doi: 10.1007/s00330-023-10568-5 (PMC11254977; doi:10.1007/s00330-023-10568-5)
Supplement: Supplementary file 1 — Supplementary file1 (PDF 140 KB) [file 330_2023_10568_MOESM1_ESM.pdf]

Diagnostic performance of 3D automated breast ultrasound (3D-ABUS) in a clinical screening setting- a retrospective study

Electronic Supplementary Material

Supplementary table: Recalls due to abnormality detected on the 3D-ABUS and/or DBT.

Detection of abnormality	Targeted HHUS immediately (N(%))	Targeted HHUS at another date (N(%))	Recalled for MRI (N(%))	Total (N(%))
3D-ABUS Alone (N(%))	**119 (3.9%)**	**65 (1.8%)**	**1 (0.1%)**	**185 (6.1%)**
3D-ABUS and DBT (N(%))	**46 (1.3%)**	**20 (0.6%)**	**3 (0.1%)**	**69 (2.3%)**
DBT Alone (N(%))	**36 (1.0%)**	**10 (0.3%)**	**2 (0.1%)**	**48 (1.6%)**
Total	**201 (5.6%)**	**95 (2.6%)**	**6 (0.2%)**	**302**

[*] 2 screening cases who underwent MRI because the mammography and 3D-ABUS were inconclusive were excluded.
